# Mutation Spectrum of Hemoglobinopathies in Tunisia

**DOI:** 10.1155/humu/6126372

**Published:** 2026-05-28

**Authors:** Imen Moumni, Khouloud Khalfaoui, Mariem Chebbi, Leila Chaouch, Houyem Ouragini, Dorra Chaouechi, Fethi Mellouli, Monia Ouederni, Monia Benkhaled, Salem Abbes, Samia Menif

**Affiliations:** ^1^ Laboratory of Molecular and Cellular Hematology (HMC), Institut Pasteur de Tunis, University of Tunis Elmanar, Tunis, Tunisia, pasteur.tn; ^2^ Department of Pediatrics: Immunology, Hematology, and Stem Cell Transplantation Bone Marrow Transplant Center, Tunis, Tunisia

**Keywords:** *β*-thalassemia (thal), delta-globin, Hb variant, hemoglobinopathies

## Abstract

**Introduction:**

Hemoglobinopathies are the most prevalent recessive disorders worldwide, characterized by wide molecular and clinical heterogeneity. They result from mutations within the alpha‐, beta‐, or delta‐globin gene leading to aberrant expression or protein function. These abnormalities are widely spread throughout the Mediterranean basin. Due to their severity and disabling nature, hemoglobinopathies represent a major public health problem. Mutational screening of abnormal hemoglobins (Hbs) allows the creation of a mutation map, which forms the basis for a potential national prevention program in Tunisia.

**Methods:**

A total of 260 Tunisian subjects were investigated using biochemical and molecular analyses to identify defects in the globin genes (HBA, HBB, and HBD). The study is aimed at determining the molecular spectrum of hemoglobinopathies and contributing to the development of a comprehensive Hb mutation map in Tunisia.

**Results:**

Twenty‐one *β*‐thalassemia mutations and 16 rare Hb variants were reported, affecting the HBA, HBD, and HBB genes. Rare Hb variants were identified and described for the first time among Tunisian patients, including Hb A_2_‐Babinga (HBD:c.410G>A; delta 136: Gly → Asp, GGT → GAT). Other variants, such as Hb Knossos, Hb Summer Hill, Hb Hope, and Hb Köln, which are uncommon in the Mediterranean region, were also detected.

**Conclusion:**

This study provides an updated overview of *β*‐thalassemia mutations and rare Hb variants in the Tunisian population. These findings are essential for understanding genetic and clinical diversity, improving the clinical management of hemoglobinopathies, and enhancing public health initiatives.

## 1. Introduction

Hemoglobinopathies are the most common group of autosomally recessively inherited monogenic disorders of hemoglobin and pose a significant health burden in the world [1]. These hemoglobin disorders are characterized by either the reduced synthesis of one or more normal globin chains, the thalassemia, and the synthesis of a structurally abnormal globin chain, the hemoglobin variants, or in a few cases by both phenotypes [2]. They are the commonest single‐gene disorders known, and approximately 1800 different mutant alleles have now been characterized by molecular analysis [3]. The mutations are regionally specific with each country having its own unique spectrum of abnormal hemoglobin and thalassemia mutations [4].

In Tunisia, because of its geographical location, it has been populated by various ethnicities. Consequently, there is considerable heterogeneity in the genetic structure of the Tunisian population [5]. Previous epidemiological studies showed the identification of a large spectrum of mutations causing hemoglobinopathies, and the disorder is found in all parts of the country [6, 7]. To date, over 28 mutations affecting the *β*‐globin gene and leading to the *β*‐thalassemia phenotype have been reported in Tunisia. The two most frequent mutations Cd39 C>T and IVS‐I‐110 G>A accounted for more than 70% of all cases [8]. For the hemoglobin variants, the best known are Hb S (HBB:c.20A>T [p.Glu7Val]) causing sickle cell disease, Hb O‐Arab (HBB:c.364G>A [p.Glu122Lys]), Hb D‐Punjab (HBB:c.364G>C [p.Glu122Gln]), and Hb C (HBB:c.19G>A [p.Glu7Lys]) [5, 6, 9, 10].

Thus, there are many carriers of hemoglobinopathies with severe clinical forms being diagnosed every year, but sufficient treatment has not yet been developed. The most effective way is to prevent the birth of hemoglobinopathy carriers through the establishment of a prevention program for hereditary blood diseases by creating national registries for healthcare systems. This is very important for genetic counseling of couples at risk and suggesting prenatal diagnosis [11, 12].

Therefore, the absence of national registries and prevention programs in our country makes it essential to perform a national program for the prevention of hereditary blood diseases by creating national registries. These programs play a critical role in managing patients with hemoglobinopathies in Tunisia. These initiatives are essential for improving public health outcomes, enhancing disease management, and facilitating research.

The aim of our work is to outline a mutational screening approach by the molecular exploration of all patients suspected of hemoglobin disorders presented in our laboratory. In addition, we elaborate an updated review of the mutation map of hemoglobinopathy mutations and rare hemoglobin variants detected in the Tunisian population.

## 2. Patients and Methods

Two hundred and sixty Tunisian subjects were referred to our laboratory for Hb investigations and molecular diagnosis between 2001 and 2021. After obtaining ethical approval from the National Ethics Committee of Tunisia (the reference: 2018/11LR16IPT07/V1) and after written informed consent was obtained, blood samples of patients were collected. Hematological indices were obtained using an automated blood cell counter (Coulter Counter ABX Micro‐60‐OTR; ABX Diagnostics, Montpelier, France). Hemoglobin pattern was performed by capillary electrophoresis using the CAPILLARYS automated system (CAPILLARYSTM 2 Sebia FLEX Piercing). Genomic DNA was isolated from peripheral blood leukocytes by the phenol/chloroform extraction method. Polymerase chain reaction (PCR) was used to amplify different fragments corresponding to the specific regions of the genes of interest (*α*‐, *β*‐, or *δ*‐globin), using respective pairs of primers [13–15]. DNA sequencing was performed on an ABI Prism 310 Genetic Analyzer Applied Biosystems, Foster City, CA, using a BigDye Terminator Sequencing Ready Reaction Kit.

## 3. Results

Among the 260 subjects diagnosed through molecular and biochemical hemoglobin screening (2001–2022), 195 patients were found to be carriers of hemoglobinopathies: 169 patients presented a beta‐thalassemia trait, and 26 patients had a rare hemoglobin variant.

### 3.1. *β*‐Thalassemia Mutations

A total of 325 beta‐thalassemia alleles from 109 homozygous and 60 compound heterozygous beta‐thalassemia patients were included in our study. Among 109 homozygous beta‐thalassemia cases, 83 patients presented with *β*°/*β*° genotype and 26 patients presented *β* ^+^/*β* ^+^genotype. However, among 60 compound heterozygous beta‐thalassemia patients, 45 cases are homozygous or compound heterozygous beta‐thalassemia (*β* ^+^/*β* ^+^, *β*°/*β*°, or *β* ^+^/*β*°), whereas only 15 patients are carriers of a beta‐thalassemia/Hb variant (Table 1).

**Table 1:** Distribution of *β*‐thalassemia genotypes in the study cohort.

Genotypes	*N* (%)	Type of *β*‐thal
Homozygous beta‐thalassemia		
*β* ^cd39(C>T)^/*β* ^cd39(C>T)^	71 (42)	*β*°/*β*°
*β* ^cd30(G>C)^/*β* ^cd30(G>C)^	2 (1.2)	*β*°/*β*°
*β* ^IVS-I-1(G>A)^/*β* ^IVS-I-1(G>A)^	2 (1.2)	*β*°/*β*°
*β* ^cd 25/26(+T)^/*β* ^cd25/26(+T)^	2 (1.2)	*β*°/*β*°
*β* ^cd106/107(+G)^/*β* ^cd106/107(+G)^	1 (0.6)	*β*°/*β*°
*β* ^cd44(del −c)^/*β* ^cd44(del −c)^	1 (0.6)	*β*°/*β*°
*β* ^cd47(+A)^/*β* ^cd47(+A)^	1 (0.6)	*β*°/*β*°
*β* ^IVS-I-2(T>G)^/*β* ^IVS-I-2(T>G)^	1 (0.6)	*β*°/*β*°
*β* ^cd6(−A)^/*β* ^cd6(−A)^	1 (0.6)	*β*°/*β*°
*β* ^IVS-II-849(A>C)^/*β* ^IVS-II-849(A>C)^	1 (0.6)	*β*°/*β*°
*β* ^IVS-I-110(G>A)^/*β* ^IVS-I-110(G>A)^	25 (14.8)	*β* ^+^/*β* ^+^
*β* ^nclt(−56)(G>C)^/*β* ^nclt(−56)(G>C)^	1 (0.6)	*β* ^+^/*β* ^+^

Compound heterozygous beta‐thalassemia		
*β* ^cd39(C>T)^/*β* ^IVS-I-110(G>A)^	12 (7.1)	*β*°/*β* ^+^
*β* ^cd39(C>T)^/*β* ^IVS-I-2(T>G)^	6 (3.5)	*β*°/*β*°
*β* ^cd39(C>T)^/*β* ^cd6(A>T)^	5(3)	*β*°/HbS
*β* ^cd39(C>T)^/*β* ^cd44(−C)^	3 (1.8)	*β*°/*β*°
*β* ^cd39(C>T)^/*β* ^cd6(−A)^	3 (1.8)	*β*°/*β*°
*β* ^cd39(C>T)^/*β* ^IVS-I-5(G>A)^	2 (1.2)	*β*°/*β* ^+^
*β* ^cd39(C>T)^/*β* ^HbC(G>A)^	2 (1.2)	*β*°/HbC
*β* ^cd39(C>T)^/*β* ^cd27(G>T)^	2 (1.2)	*β*°/*β* (Hb Knossos)
*β* ^cd39(C>T)^/*β* ^IVS-I-1(G>A)^	2 (1.2)	*β*°/*β*°
*β* ^cd39(C>T)^/*β* ^cd30(G>C)^	1 (0.6)	*β*°/*β*°
*β* ^cd39(C>T)^/*β* ^IVS-II-16(G >C)^	1 (0.6)	*β*°/*β* ^+^
*β* ^cd39(C>T)^/*β* ^cd5(−CT)^	1 (0.6)	*β*°/*β*°
*β* ^cd39(C>T)^/*β* ^IVS-I-6(T>C)^	1 (0.6)	*β* ^+^/*β*°
*β* ^cd39(C>T)^/*β* ^IVS-II-745(C>G)^	1 (0.6)	*β*°/*β* ^+^
*β* ^IVS-I-110(G>A)^/*β* ^cd30(G>C)^	2 (1.2)	*β* ^+^/*β*°
*β* ^IVS-I-110(G>A)^/*β* ^IVS-I-5(G>C)^	1 (0.6)	*β* ^+^/*β* ^+^
*β* ^IVS-I-110(G>A)^/*β* ^IVS-I-6(T>C)^	3 (1.8)	*β* ^+^/*β* ^+^
*β* ^IVS-I-110(G>A)^/*β* ^cd44(−C)^	2 (1.2)	*β* ^+^/*β*°
*β* ^IVS-I-110(G>A)^/*β* ^cd6(A>T)^	3 (1.8)	*β* ^+^/HbS
*β* ^IVS-I-1(G>A)^/*β* ^IVS-I-6(T>C)^	1 (0.6)	*β*°/*β* ^+^
*β* ^IVS-I-2^/*β* ^cd6(−A)^	1 (0.6)	*β*°/*β*°
*β* ^IVS-II-745(C>G)^/*β* ^cd6(A>T)^	1 (0.6)	*β* ^+^/HbS
*β* ^IVS-I-1(G>A)^/*β* ^cd6(A>T)^	1(0.6)	*β*°/HbS
*β* ^cd8(−AA)^/*β* ^cd6(A>T)^	1 (0.6)	*β*°/HbS
*β* ^cd30(G>C)^/*β* ^polyA(T>A)^	1 (0.6)	*β*°/*β* ^+^
*β* ^nclt(−29)(A>G)^/*β* ^cd44(−C)^	1 (0.6)	*β* ^+^/*β*°
Total	169	

The molecular analysis of 325 beta‐thalassemia alleles revealed that 21 *β*‐thalassemia mutations have been described to date in Tunisia (Tables 1 and 2). The molecular data show that the Codon 39 HBB:c.118C>T (p.Gln40Ter), rs11549407 and IVS‐I‐110 HBB:c.93‐21G>A (p.?), rs35004220 mutations are the most common *β*‐thalassemia defects in Tunisia (56% and 22%, respectively) (Table 2). They are followed, in decreasing order, by IVS‐I‐2 [HBB:c.92+2T>G (p.?), rs33956879], IVS‐I‐1 [HBB:c.92+1G>A (p.?), rs33971440], Hb Kairouan ou Codon 30 [HBB:c.92G>C (p.Arg31Thr), rs33960103], IVS‐I‐6 [HBB:c.92+6T>C (p.?), rs35724775], IVS‐I‐5 [HBB:c.92+5G>A (p.?), rs33915217], Codon 25/26 (+T) [HBB:c.78_79insT (p.Leu26ProfsTer15), rs35619688], Codon 44 (−C) [HBB:c.135delC (p.Pro46LeufsTer14), rs80356820], Codon 6 (−A) [HBB:c.20delA (p.Lys7ArgfsTer11), rs63749819], nucleotide −29 [HBB:c.‐79A>G, rs34598529], Codon 106/107 [HBB:c.321_322insG (p.Gly108ArgfsTer31), rs35225141], Hb Knossos Codon 27 [HBB:c.82G>T (p.Ala28Ser), rs35424040], Codon 47 [HBB:c.143_144insA (p.Glu48ValfsTer4), rs35894115], IVS‐II‐849 [HBB:c.316‐2A>C (p.?), rs33914668], IVS‐I‐5 [HBB:c.92+5G>C (p.?), rs33915217], Codon 5[HBB:c.17_18del (p.Pro6ArgfsTer17), rs3488988], IVS‐II‐16 [HBB:c.315+16G>C, rs10768683], IVS‐II‐745 [HBB:c.316‐106C>G, rs34690599], Poly A [HBB:c.∗110T>A (Poly A signal: AATAAA>AAAAAA)], Codon 8 [HBB:c.25_26del (p.Lys9ValfsTer14), rs35497102], nucleotide −56 [HBB:c.‐106G>C, rs63750681], and nucleotide −83 [HBB:c.‐133G > A, rs72561473].

**Table 2:** The frequency of beta‐thalassemia mutations in 325 alleles.

Alleles	Number of alleles (%)
Codon 39 (C>T)	184 (56.8)
IVS‐I‐110 (G>A)	73 (22.5)
IVS‐I‐2 (T>G)	9 (2.8)
Codon 44 (del −c)	8 (2.5)
Codon 30 (G>C)	8 (2.5)
IVS‐I‐1 (G>A)	8 (2.5)
Codon 6 (−A)	6 (1.9)
IVS‐I‐6 (T>C)	5 (1.5)
Codon 25/26 (+T)	4 (1.2)
IVS‐I‐5 (G>A)	3 (0.9)
Codon 106/107 (+G)	2 (0.6)
Codon 47 (+A)	2 (0.6)
IVS‐II‐849 (A>C)	2 (0.6)
IVS‐II‐745 (C>G)	2 (0.6)
Nucleotide (−56) (G>C)	2 (0.6)
Codon 27 (G>T) Hb Knossos	2 (0.6)
IVS‐II‐16 (G>C)	1 (0.3)
Codon 5 (−CT)	1 (0.3)
Codon 8 (−AA)	1 (0.3)
Poly A (T>A)	1 (0.3)
Nucleotide (−29) (A>G)	1 (0.3)
Total	325

Table 1 presents the distribution of various beta‐thalassemia genotypes among 169 patients in Tunisia, categorized into two main groups: the homozygous beta‐thalassemia genotypes (°/° and ^+^/^+^) and the compound heterozygous beta‐thalassemia (°/° or ^+^/^+^ or °/^+^).

The homozygous beta‐thalassemia (°/°) accounts for the largest proportion with 81 patients (47.9%). The most frequent mutation is ^cd39(C>T)^/^cd39(C>T)^, present in 71 patients (42%), indicating a predominant founder effect or mutation hotspot. The other homozygous mutations are rare such as ^cd44(del −c)^/^cd44(del −c)^, contributing ≤ 1% of cases. Regarding the homozygous ^+^/^+^ genotype, observed in 26 patients (15.4%), the predominant genotype is ^IVS-I-110(G>A)^/^IVS-I-110(G>A)^, found in 25 patients (14.8%), suggesting it as a common mild mutation in this population.

The compound heterozygous beta‐thalassemia group includes 62 patients (36.7%), demonstrating considerable genetic heterogeneity. The most frequent combinations include ^cd39(C>T)^/^IVS-I-110(G>A)^ 12 patients (7.1%), ^cd39(C>T)^/^IVS-I-2(T>G)^ 6 patients (3.5%), and ^cd39(C>T)^/^cd6(A>T)^ (HbS) 5 patients (3%). Multiple combinations observed in our cohort include interactions with HbS and HbC, pointing to beta‐thalassemia/hemoglobinopathy coinheritance.

Table 2 summarizes *β*‐thalassemia allele counts; therefore, Hb Knossos (HBB:c.82G>T) was included as a *β*‐thalassemic allele, whereas the corresponding carrier is described separately in Table 3.

**Table 3:** Hemoglobin gene variants found in the Tunisian population.

HGVS nomenclature	Amino acid substitution	Name	Genotype	Variant clinical/hematological significance	*N*
HBA2:c.207C>A	Alpha2 68(E17) Asn>Lys	Hb G‐Philadelphia	*α* ^cd68 AAC → AAA^ *α*/*αα*	Benign	2
HBA2:c.427T>C	Alpha2 142, Stop>Gln	Hb Constant Spring	*α* ^cd142 TAA → CAA^ *α*/*αα*	Hemolytic anemia	1
HBB:c.23A>G	Beta 7(A4) Glu>Gly	Hb G‐San Jose	*β* ^cd7GAG → GGG^/*β* ^cd39CAG → TAG^	Benign	1
HBB:c.170G>A	Beta 56(D7) Gly>Asp	Hb J‐Bangkok	*β* ^cd56GGC → GAC^/*β*	Benign	2
HBB:c.295G>A	Beta 98(FG5) Val>Met	Hb Köln	*β* ^cd98GTG → ATG^/*β*	Mild hemolytic Anemia	1
HBB:c.410G>A	Beta 136(H14) Gly>Asp	Hb Hope	*β* ^cd136GGT → GAT^/*β*	Benign	1
HBB:c.157G>C	Beta 52(D3) Asp>His	Hb Summer Hill	*β* ^cd52GAT → CAT^/*β*	Benign	1
HBB:c.67G>C	Beta 22(B4) Glu>Gln	Hb D‐Iran	*β* ^cd22GAA → CAA^/*β*	Benign	3
HBB:c.122G>A	Beta 40(C6) Arg>Lys	Hb Athens‐Georgia	*β* ^cd40AGG → AAG^/*β*	Benign	1
HBB:c.82G>T	Beta 27(B9) Ala>Ser	Hb Knossos	*β* ^cd27GCC → TCC^/*β* ^cd39CAG → TAG^	*β* ^+^ mutation, Moderate Anemia	1
HBB:c.364G>C	Beta 121(GH4) Glu>Gln	Hb D‐Punjab	*β* ^cd21GAA → CAA^/*β*	Benign	1
HBD:c.140G>A	Delta 46(CD5) Gly>Glu	Hb A_2_‐Tunis	*δ* ^cd46GGG → GAG^/*β* ^IVS-I-1(G → A)^	NA	1
HBD:c.49G>C	Delta 16(A13) Gly>Arg	Hb A_2_ ^′^	*δ* ^cd16GGC → CGC^/*β* ^cd39CAG → TAG^	NA	3
HBD:c.82G>T	Delta 27(B9) Ala>Ser	Hb A_2_‐Yialousa	*δ* ^cd27GCC → TCC^/*β*	Delta^+^, Benign	2
			*δ* ^cd27GCC → TCC^/*δ* ^cd27GCC → TCC^		1
			*δ* ^cd27GCC → TCC^/*β* ^IVS-I-1(G → A)^		1
HBD:c.410G>A	Delta 136(H14) Gly>Asp	Hb A_2_‐Babinga	*δ* ^cd136 GGT → GAT^/*β*	NA	1
			*δ* ^cd136 GGT → GAT^/*β*‐thal		1
HBD:c.180G>C	Delta 59(E3) Lys>Asn	Hb A_2_‐Pasteur‐Tunis	*δ* ^cd59AAG → AAC^/*β* ^cd39CAG → TAG^	Benign	1
Total					26

*Note:* The information reported in the column “Variant’s clinical/hematological significance” was annotated using data from the IthaNet (https://www.ithanet.eu/) and HbVar (https://globin.bx.psu.edu/hbvar/menu.html) databases.

Abbreviation: NA, not available.

### 3.2. Rare Hemoglobin Variants

Table 3 summarizes 16 hemoglobin variants identified in 26 individuals from the Tunisian population. These include mutations in the alpha‐globin (*HBA2*), beta‐globin (*HBB*), and delta‐globin (*HBD*) genes, each leading to structurally abnormal hemoglobins or rare hemoglobin variants. The variants represent both globally known hemoglobinopathies and mutations with regional or potentially local specificity.

#### 3.2.1. Alpha‐Globin Variant

The *α*2‐globin gene (*HBA2*) mapping reveals two uncommon mutations: Hb G‐Philadelphia (HBA2:c.207C>A [p.Asn69Lys], rs1060339) and Hb Constant Spring (HBA2:c.427T>C [p.Ter143GlnextTer31], rs41464951), among three Tunisian cases. The two cases (sex: male/age: 10 and 12) with Hb G‐Philadelphia present a microcytic anemia (RBC = 6.5 10^12^/L, Hb = 10.4 g/dL, Ht = 32.7*%*, MCV = 58.7FL, MCH = 18.7Pg) and displayed two abnormal Hb fractions (27.5%) in the electrophoresis pattern: a major one migrating close to Hb S (negative solubility test) and a minor one migrating behind the normal Hb A_2_ (2.31%). DNA analysis of the *α*2‐globin gene confirms the presence of a mutation Hb G‐Philadelphia (HBA2:c.207C>A [p.Asn69Lys]) (Table 3). The Hb Constant Spring carrier (sex: male/age: 19) presents an HbH fraction in the electrophoresis pattern and a microcytic anemia (RBC = 5.6 10^12^/L, Hb = 9.6 g/dL, MCV = 65FL, MCH = 17.8Pg). However, DNA sequencing of the *α*2‐globin gene revealed a mutation at 142 amino acids characterizing the Hb Constant Spring (HBA2:c.427T>C [p.Ter143GlnextTer31], rs41464951) (Table 3).

#### 3.2.2. Delta‐Globin Variants

The *δ*‐globin gene (*HBD*) mapping reveals five uncommon mutations among 11 patients: Hb A_2_ ^′^ or Hb B_2_ [HBD:c.49G>C (p.Gly17Arg)], Hb A_2_‐Yialousa [HBD:c.82G>T (p.Ala28Ser)], Hb A_2_‐Tunis [HBD:c.140G>A (p.Gly47Glu)], Hb A_2_‐Babinga [HBD:c.410G>A (p.Gly137Asp)], and Hb A_2_‐Pasteur‐Tunis [*δ*59 Lys → Asp; AAG → AAC HBD:c.180G>C (p.Lys60Asn)] (Table 3).

**Figure 1:**
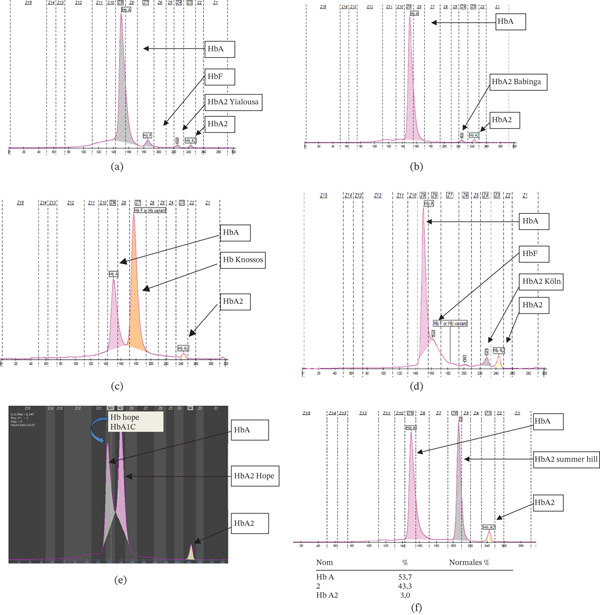
Capillary electrophoresis profiles. (a) Case carrying the Hb variant Hb A_2_‐Yialousa. (b) Case carrying the Hb variant Hb A_2_‐Babinga. (c) Case carrying the Hb variant Hb Knossos. (d) Case carrying the Hb variant Hb Köln. (e) Case carrying the Hb variant Hb Hope. (f) Case carrying the Hb variant Hb Summer Hill.

HbA_2_‐Yialousa was detected in four Tunisian patients. The two first cases (sex: female/age: 12 and 30) present a microcytic anemia (RBC = 5.6 10^12^/L, Hb = 11 g/dL, Ht = 37*%*, MCV = 75FL, MCH = 29Pg) and a low level of Hb A2 (1.9%) (Figure 1a). DNA analysis of the *δ*‐globin gene revealed a mutation at Codon 27 leading to Hb A_2_‐Yialousa [*δ*27 Ala → Ser; GCC → TCC] and a *δ*°‐thalassemia heterozygote phenotype. The second case (sex: female/age: 27) presents a microcytic anemia (RBC = 5 10^12^/L, Hb = 11.5 g/dL, Ht = 37.5*%*, MCV = 76FL, MCH = 30Pg) and nonappearance of Hb A2. DNA analysis revealed a homozygous genotype (*δ*°/*δ*°) of Hb A_2_‐Yialousa. The third case (sex: female/age: 17) presents also a microcytic anemia (RBC = 5 10^12^/L, Hb = 10 g/dL, Ht = 37*%*, MCV = 67FL, MCH = 29Pg) and a low level of Hb A2 (0.8%). DNA analysis revealed a compound heterozygosis genotype *δ*°‐Hb A_2_‐Yialousa and *β*°‐IVS‐I‐1 (G>A). In this case, Hb A_2_‐Yialousa has been described in association with *β*°‐thalassemia (Table 3).

HbA_2_‐Babinga was detected in two Tunisian patients. The first case (sex: female/age: 38) presents normal hematological and biochemical indices (RBC = 4.07 10^12^/L, Hb = 13 g/dL, Ht = 38.9*%*, MCV = 95.6FL, MCH = 31.729Pg) and a low level of Hb A2 (1.1%) (Figure 1b), whereas electrophoresis analysis shows fast‐moving minor HbX (0.8%) fraction moving in front of the normal Hb A2 fraction. DNA analysis shows a heterozygote mutation at Codon 136 of the delta gene leading to HbA_2_‐Babinga HBD:c.410G>A (p.Gly137Asp), rs35849348 (Table 3).

The second case (sex: female/age: 45) presents hematological *β*‐thalassemia trait, contrasting with a normal Hb A2 level and the presence of HbF = 4.1*%* (RBC = 4.86 10^12^/L, Hb = 10.5 g/dL, Ht = 32.4*%*, MCV = 66.7FL, MCH = 21.6Pg, Hb A2 = 1.9*%*, and HbX = 1.3*%*). DNA analysis of the *δ*‐globin gene revealed a compound heterozygosis genotype HbA_2_‐Babinga/*β*°‐thalassemia Codon 39 HBB:c.118C>T (p.Gln40Ter), rs11549407 (Table 3).

Hb A2 ^′^ or Hb B2 was detected among one case (sex: male/age: 4 years), which presenting a *β*‐thalassemia trait despite with normal Hb A2 level (RBC = 5.7 10^12^/L, Hb = 11.9 g/dL, Ht = 36.5*%*, MCV = 64.1FL, MCH = 20.8Pg, Hb A2 = 1.95*%*, and HbX = 2.4*%*). DNA sequencing of the delta and beta‐globin gene reveals a compound heterozygosis genotype Hb A2 ^′^ or Hb B2 HBD:c.49G>C (p.Gly17Arg) and *β*°‐thalassemia Codon 39 HBB:c.118C>T (p.Gln40Ter), rs11549407 (Table 3).

Seven patients carrying HBD variants have been reported in association with the *β*‐thalassemia trait (Table 3). At the time of diagnosis, all individuals revealed hematological profiles consistent with *β*‐thalassemia, including microcytosis and hypochromia, accompanied by the presence of an additional hemoglobin peak (HbX) and a normal HbA2 level (Table 3). These findings align with our results, reinforcing those rare delta‐globin variants that can hide the typical *β*‐thalassemia diagnostic pattern. Specifically, they can normalize HbA2 levels, leading to potential misdiagnosis or underdiagnosis of *β*‐thalassemia, especially in asymptomatic carriers.

#### 3.2.3. Beta‐Globin Variant

The DNA analysis results of the *β*‐globin gene revealed nine abnormal Hb variants: Hb G‐San Jose, Hb D‐Iran, Hb Knossos, Hb Summer Hill, Hb J‐Bangkok, Hb Köln, Hb D‐Punjab, Hb Hope, and Hb Athens‐Georgia.

Twelve cases with different phenotypes were spotted for an unknown capillary electrophoretic profile. Hb G‐San Jose was identified in a Tunisian family, investigated for *β*‐thalassemia. The capillary electrophoresis analysis showed a compound heterozygote patient with *β*‐thalassemia, and an abnormal Hb variant migrating slightly ahead of Hb S. DNA sequencing of the *β*‐globin gene revealed two mutations: *β*°‐thalassemia HBB:c.118C>T (p.Gln40Ter) and Hb G‐San Jose (Table 3).

Hb J‐Bangkok was detected among two patients (sex: female/age: 43 and 62). Electrophoresis analysis displayed a fast‐moving band migrating ahead of HbA, despite normal hematological findings (RBC = 4.5 10^12^/L, Hb = 13.6 g/dL, Ht = 41.5*%*, MCV = 91.51FL, MCH = 29.9Pg, Hb A2 = 2.5*%*, and HbX = 53*%*) (Table 3). DNA analysis of the *β*‐globin gene showed a mutation Hb J‐Bangkok [beta 56(D7); Gly>Asp; HBB: c.170G>A].

Hb Knossos found among patients (sex: male/age: 20) presents a microcytic hypochromic anemia (RBC = 5.8 10^12^/L, Hb = 11.5 g/dL, Ht = 35.6*%*, MCV = 58.5FL, MCH = 27Pg, Hb A2 = 1.2*%*, and HbX = 66.5*%*). Capillary electrophoretic investigations showed a compound heterozygote patient with *β*‐ thalassemia and an abnormal Hb variant migrating slightly ahead of Hb A (Figure 1c). DNA analysis of the *β*‐globin gene revealed two mutations: Hb Knossos [beta 27(B9); Ala>Ser; HBB:c.82G>T] and *β*°‐thalassemia [beta 39(C5), Gln>Stop; HBB:c.118C>T]. Hb Knossos is a cause of misdiagnosis in *β*‐thalassemia, as it results in a normal Hb A2 level, similar to that seen in asymptomatic individuals. Despite carrying a *β* ^0^‐thalassemia mutation, patients with Hb Knossos exhibit an intermediate *β*‐thalassemia phenotype, which can lead to misdiagnosis (Table 3).

Hb Köln patient was detected among patients (sex: female/age: 18) with severe normocytic anemia and slightly hypochromia (RBC = 5.66 10^12^/L, Hb = 9 g/dL, Ht = 35*%*, MCV = 79FL, MCH = 25Pg, Hb A2 = 5.7*%*, and HbX = 5.4*%*). Hemoglobin investigations show small migrating peaks between Hb A and Hb A2 zones, and the stability test revealed unstable hemoglobin (Figure 1d). Molecular analysis of the *β*‐globin gene revealed a mutation leading to the beta unstable variant known as Hb Köln [beta 98(FG5); Val>Met; HBB:c.295G>A] (Table 3).

Hb Hope was revealed among patients (sex: male/age: 40) with normal hematological and biochemical indices (RBC = 5.3 10^12^/L, Hb = 13 g/dL, Ht = 35.2*%*, MCV = 80FL, MCH = 32Pg). However, capillary electrophoretic reports a normal value for the Hb A2 fraction (2.9%) but had an additional peak HbX (35%) close to HbA (Figure 1e). *β*‐Globin gene sequencing reveals the beta variant: Hb Hope [beta 136(H14); Gly>Asp; HBB:c.410G>A] (Table 3).

Hb Summer Hill was revealed among patients (sex: male/age: 5 years) with normal hematological indices with microcytic (RBC = 5.2 10^12^/L, Hb = 12.8 g/dL, Ht = 37*%*, MCV = 77FL, MCH = 30Pg, Hb A2 = 3*%*, and HbX = 43.3*%*) (Figure 1f). The capillary electrophoresis shows the *β* variant S‐Like, with a negative solubility test. Molecular analysis of the *β*‐globin gene revealed the beta variant: Hb Summer Hill: [HBB beta 52(D3); Asp>His; HBB:c.157G>C] (Table 3).

Hb Athens‐Georgia was also revealed among patients (sex: female/age: 30) with normal hematological indices (RBC = 5.7 10^12^/L, Hb = 12.5 g/dL, Ht = 35*%*, MCV = 81FL, MCH = 32.5Pg). The capillary electrophoresis shows the *β* variant close to normal HbA (HbX = 45.5*%*). The *β*‐globin gene analysis reveals the Hb Athens‐Georgia: [beta 40(C6); Arg>Lys; HBB:c.122G>A].

## 4. Discussion

This study provides a comprehensive molecular characterization of hemoglobinopathies in Tunisia, revealing a diverse mutation spectrum that reflects the country’s unique genetic heritage and epidemiological profile. The analysis of 325 *β*‐thalassemia alleles and the identification of 16 rare hemoglobin variants offer critical insights into the genetic background of these disorders in a Mediterranean population shaped by historical migrations, high consanguinity, and ethnic diversity [5]. Below, we discuss the key findings in the context of mutation prevalence, rare variant significance, diagnostic challenges, clinical implications, and public health strategies, while establishing comparisons with regional and global data.

### 4.1. Prevalence and Distribution of *β*‐Thalassemia Mutations

Beta‐globin gene mutation screening is crucial for the prevention and management of *β*‐thalassemia and other hemoglobin disorders. It plays an important role in molecular diagnosis, genetic counseling, and the development of targeted therapies. In Tunisia, it presents significant insights into the genetic background of the hemoglobin disorder within our population.

In our prospective cohort, we identified 21 *β*‐thalassemia mutations among 325 analyzed *β*‐thalassemia alleles from 109 homozygous and 60 compound heterozygous patients, revealing a highly molecular heterogeneous mutational pattern, both in prevalence and genotype distribution.

This study of 169 Tunisian patients with *β*‐thalassemia genotypes highlights both the high prevalence of severe mutations and the genetic diversity of *β*‐thalassemia in Tunisia. The predominance of the Codon 39 [beta 39(C5); Gln > Stop] mutation (present in over 60% of cases either in homozygous or compound heterozygous forms) confirms its epidemiological importance in Tunisia. This mutation is a well‐known *β*°‐thalassemia mutation commonly observed in Mediterranean populations [6, 16, 17]. The IVS‐I‐110 [beta nt 252 G>A] mutation, a mild allele (*β* ^+^‐thalassemia), is the most common cause of thalassemia intermedia and frequently appears in compound heterozygous forms. Its relatively high frequency suggests that it may have been maintained in the population through heterozygote advantage or founder effects [8].

The detection of compound heterozygous genotypes with structural hemoglobin variants (HbS and HbC) indicates that overlapping hemoglobinopathies are present in Tunisia. These combinations can complicate clinical phenotypes and pose additional challenges for diagnosis and management [9]. The presence of many rare mutations, each found in one or two individuals, underlines the genetic heterogeneity of *β*‐thalassemia in Tunisia. This diversity may reflect the ethnic and historical complexity of our country, including gene flow from sub‐Saharan Africa, the Middle East, and Europe [5]. As our data were obtained from a single diagnostic referral center, the observed frequencies reflect our cohort rather than the general Tunisian population. From a public health perspective, these findings underscore the need for comprehensive genetic screening in Tunisia for both carrier detection and prenatal diagnosis, especially focusing on the most common mutations: Codon 39 and IVS‐I‐110.

Tunisian beta‐thalassemia mutation spectrum differs from those in Mediterranean populations [18], indicating unique genetic characteristics within the Tunisian population. Our study reports newly identified mutations specific to Tunisian populations: Hb Kairouan (Codon 30), Codon 25/26 (+T), Codon 44 (−C), nucleotide (−56), nucleotide (−83), and Poly A (AATAAA>AAAAAA). This highlights that the Tunisian population has unique genetic features due to the high consanguinity (1,3) and the diverse genetic origins. Hence, it underscores the importance of the implementation of a national registry and targeted research to improve diagnosis and treatment strategies [19].

The Codon 39 (C>T) and IVS‐I‐110 (G>A) mutations are among the most prevalent *β*‐thalassemia mutations identified in Tunisia, reflecting the genetic influence of both Mediterranean and Middle Eastern populations. The Codon 39 mutation account is highly prevalent in Sardinia, where it constitutes up to 95% of cases [18]. It is moderately frequent in Spain and Greece, less common in the Middle East, and rare or absent in South and Southeast Asia [20]. The IVS‐I‐110 mutation is one of the most common mutations in Lebanon, Syria, Iraq, and Egypt, with frequencies ranging from 30% to 40% [21]. It is also frequently observed in mainland Italy and Greece but is less common in South Asia and rarely detected in Southeast Asia [22]. This highlights the distinct mutation profiles in Tunisia compared to other regions, influenced by historical migrations and genetic diversity.

The genotype analysis revealed that the most common genotypes included 76% with beta‐thalassemia (*β*°/*β*°), while 24% had beta‐thalassemia (*β* ^+^/*β* ^+^). This distribution indicates a higher incidence of severe forms of the disease among Tunisian patients, which may have implications for clinical management and genetic counseling in affected families.

The high frequency of these mutations and genotype (*β*°/*β*°) in Tunisia highlights the importance of targeted molecular screening programs and reinforces the need for region‐specific strategies in genetic counseling and prenatal diagnosis [23].

### 4.2. Rare Hemoglobin Variants

In the context of this study, the term “rare hemoglobin variants” refers to uncommon variants identified in our cohort, including variants not previously reported in Tunisia. Several rare hemoglobin variants have been identified, resulting from various mutations in the genes encoding globin chains affecting *α*‐, *δ*‐, and *β*‐globin genes, thus expanding the known mutational spectrum in Tunisia. This can have different effects on the structure and function of hemoglobin, and they can be associated with specific hemoglobinopathies or have no clinical significance. To date, the total number of rare Hb variants is over 1800, which have been reported by Hbvar [24]. In Tunisia, some sporadic cases of rare Hb variants were described, and they were observed once at the heterozygous state or in association with *α*‐ or *β*‐thalassemia, although other rare Hb variants reported are specific to the Tunisian population such as Hb Pasteur‐Tunis [9].

Our results show that 24 cases were observed to be carrying rare Hb mutations. It was identified that there were 16 mutation carriers in *α* (*HBA*), *β* (*HBB*), and *δ* (*HBD*) globin genes. Two mutations were observed in the *HBA2* gene: Hb G‐Philadelphia and Hb Constant. Nine mutations were observed in the *HBB* gene: Hb G‐San Jose, Hb D‐Iran, Hb Knossos, Hb Summer Hill, Hb J‐Bangkok, Hb Köln, Hb D‐Punjab, Hb Hope, and Hb Athens‐Georgia. Five mutations were observed in the *HBD* gene: Hb A_2_ ^′^ or Hb B_2_, Hb A_2_‐Tunis, and HbA_2_‐Pasteur‐Tunis, and two mutations were described for the first time in Tunisia: Hb A_2_‐Yialousa and Hb A_2_‐Babinga. These findings underscore both the genetic heterogeneity of hemoglobinopathies in Tunisia and the multiethnic origins of Tunisians.

In the Tunisian population, Hb G‐Philadelphia, although rare [7], is consistent with historical genetic admixture from sub‐Saharan Africa and the Mediterranean basin [25]. Its relatively high frequency in African populations and occasional occurrence in North Africa suggest an ancient gene flow into the region, likely facilitated by trade and migration. Hb Constant Spring, on the other hand, is a hallmark of Southeast Asian *α*‐thalassemia [26] and is rarely encountered in Tunisia. Its detection may be explained by rare migration events or de novo mutations [7].

The *HBB* variants are globally recognized; their frequency in Tunisia is extremely low or absent, except for Hb D‐Punjab and Hb D‐Iran, which may appear as sporadic cases. Hb D‐Punjab is the most frequent due to its wider geographic distribution in North Africa [27]. Hb D‐Iran and Hb Knossos reflect historical and geographical connections between Tunisia and the broader Mediterranean and Middle Eastern regions [28]. Hb Knossos is of particular interest because it can lead to misdiagnosis of *β*‐thalassemia when coinherited with other mutations. It is a rare *HBB* variant, causing *β* ^+^‐thalassemia, more commonly reported in Greece [29]. In Tunisia, its presence is frequently in compound heterozygosis with *β*°‐thalassemia, illustrating how rare HBB variants can modify disease severity, producing an intermediate phenotype despite a *β*° mutation [29]. Hb Summer Hill, Hb J‐Bangkok, and Hb Köln are mostly endemic to Southeast Asia or Europe and are absent from Tunisian studies, suggesting no local circulation [9, 30–32]. In our cohort, Hb J‐Bangkok was detected at a relatively high proportion on hemoglobin analysis despite normal hematological indices, supporting its limited clinical significance and likely structural stability. Hb G‐San Jose, Hb Hope, and Hb Athens‐Georgia have been sporadically reported globally and may be found only in highly specific contexts such as admixture or migration.

Three *HBD* variants described in our study are specific to our population: Hb A_2_ ^′^ or Hb B_2_, Hb A_2_‐Tunis, and HbA_2_‐Pasteur‐Tunis [9]. Hb A_2_‐Yialousa and Hb A_2_‐Babinga are the first descriptions in African populations [33], and Hb A_2_‐Yialousa has been reported in Greece and Cyprus, regions with a high *β*‐thalassemia prevalence [6]. Hb A_2_‐Babinga, originally described in Central Africa, illustrates how gene flow through migration or historical trade routes may explain its presence in Tunisia.

Our results indicate a high molecular heterogeneity of the hemoglobin genes, which is likely due to our country’s strategic geographic position, as it has historically facilitated gene flow between Europe, the Middle East, and sub‐Saharan Africa [4]. These findings highlight the importance of ongoing molecular surveillance in Tunisia’s hemoglobinopathies prevention programs to capture the full spectrum of rare variants, especially in mixed or undiagnosed cases.

### 4.3. Diagnostic Challenges and Methodological Considerations

The coexistence of rare Hb variants and *β*‐thalassemia mutations poses significant diagnostic challenges, particularly with standard techniques like capillary electrophoresis. Electrophoresis, while effective for common variants (Hb S, Hb C, and Hb O), often fails to detect unstable variants like Hb Knossos and Hb Köln. Hb Knossos, for example, migrates near Hb A and normalizes Hb A2 levels, mimicking an asymptomatic state despite underlying *β*‐thalassemia pathology. This misdiagnosis risk is compounded in compound heterozygotes, where phenotypic severity deviates from expected patterns. Similarly, Hb Köln’s instability requires additional stability tests (isopropanol precipitation test), which are not routine in many laboratories.

Delta‐globin variants further complicate screening, as they alter Hb A2 quantification, which is a key parameter in screening for *β*‐thalassemia carriers. In our study, patients with Hb A_2_‐Babinga or Hb A_2_‐Yialousa exhibited borderline or normal Hb A2 levels despite *β*‐thalassemia traits, highlighting the limitations of relying solely on biochemical markers. These observations align with global reports of diagnostic errors in multivariant populations [7, 8, 34] and advocate for integrating DNA sequencing into routine practice. Sequencing not only resolves ambiguous electrophoretic profiles but also identifies novel mutations, as demonstrated by our detection of Tunisia‐specific alleles. However, cost and infrastructure constraints in Tunisia limit widespread adoption, underscoring the need for targeted molecular diagnostics in referral centers.

Consequently, integrating molecular diagnostics, such as sequencing at the same time as *HBB*, *HBD*, and *HBA* genes, becomes crucial in atypical cases to uncover underlying mutations that might not be evident through routine hemoglobin electrophoresis alone [35]. This approach ensures accurate diagnosis and appropriate genetic counseling.

### 4.4. Clinical and Genetic Implications

The high prevalence of severe *β*°/*β*° genotypes (76% of homozygous cases) versus milder *β* ^+^/*β* ^+^ forms (24%) reflects a substantial clinical burden in Tunisia. Patients with *β*° mutation Cd39 homozygosity typically require lifelong transfusions and chelation therapy, straining healthcare resources. In contrast, compound heterozygotes with rare variants (Hb Knossos/*β*°‐thal) exhibit variable phenotypes, ranging from transfusion independence to moderate anemia, complicating prognostic assessments. This heterogeneity necessitates individualized management plans, informed by molecular data, to optimize outcomes.

Consanguinity, prevalent in Tunisia at rates of 20%–30% [3, 22] amplifies the risk of homozygous and compound heterozygous states, increasing disease incidence. The novel mutations identified here, though rare, may segregate within specific families or regions, warranting pedigree analyses to trace their origins and penetrance. Moreover, the interplay of HbA, HBD, and HBB variants suggests potential epigenetic effects on phenotype severity. Future studies should employ functional assays, rapid sequencing by NGS, and clinical follow‐ups to elucidate these interactions, enhancing genotype–phenotype correlations.

### 4.5. Public Health and Preventive Strategies

The absence of a national hemoglobinopathy registry in Tunisia delays accurate prevalence mapping and resource allocation, a gap this study seeks to address. The mutation map presented here, dominated by Cd39 and IVS‐I‐110 mutations, yet enriched with rare variants, provides a model for targeted screening programs. Prenatal diagnosis and genetic counseling, proven effective in reducing thalassemia incidence in Cyprus and Italy [36], could be prioritized for couples carrying high‐risk genotypes. Newborn screening, coupled with carrier detection, would further enable early intervention, mitigating long‐term complications.

However, implementing such programs faces challenges: limited public awareness, cultural resistance to genetic testing, and inadequate funding. The economic burden of managing transfusion‐dependent patients, estimated at thousands of dollars annually per patient in similar settings [37–39], underscores the cost‐effectiveness of prevention over treatment. International models, such as Bahrain’s thalassemia control program, which reduced incidence by 60% through mandatory screening, offer a roadmap. Tunisia could adapt these strategies by focusing on high‐prevalence regions and leveraging existing infrastructure like the Pasteur Institute.

## 5. Conclusion

In conclusion, this comprehensive analysis of hemoglobinopathies alleles in Tunisia underscores the importance of understanding local mutation spectra for effective management of the disease. The predominance of certain mutations highlights potential areas for focused research and public health initiatives aimed at reducing the burden of beta‐thalassemia in affected populations. Future studies should incorporate next‐generation sequencing approaches to identify deep intronic or regulatory variants not detected by conventional methods and should investigate genotype–phenotype correlations in larger cohorts.

## Author Contributions

The authors alone are responsible for the content and writing of the paper.

## Funding

This project was supported by the Tunisian Ministry of Higher Education and Scientific Research, Laboratory of Molecular and Cellular Hematology (Code LR21IPT07).

## Ethics Statement

The study was conducted in accordance with the Declaration of Helsinki and was approved by the Institutional Ethics Committee, biomedical ethics committee of the Pasteur Institute of Tunis (Protocol Code 2018/11/I/LR16IPT07/V1).

## Consent

Informed consent was obtained from all participants involved in this study. Written informed consent has been obtained from the participants to publish this paper.

## Conflicts of Interest

The authors declare no conflicts of interest.

## Data Availability

The data that support the findings of this study are available on request from the corresponding author. The data are not publicly available due to privacy or ethical restrictions.
